# Investigation of Plant Antimicrobial Peptides against Selected Pathogenic Bacterial Species Using a Peptide-Protein Docking Approach

**DOI:** 10.1155/2022/1077814

**Published:** 2022-03-21

**Authors:** Ghulam Mustafa, Rizwan Mehmood, Hafiza Salaha Mahrosh, Khalid Mehmood, Shakeel Ahmed

**Affiliations:** ^1^Department of Biochemistry, Government College University Faisalabad, Faisalabad-38000, Pakistan; ^2^Department of Biochemistry, University of Agriculture Faisalabad, Faisalabad-38000, Pakistan; ^3^Faculty of Veterinary and Animal Sciences, The Islamia University of Bahawalpur, Bahawalpur-63100, Pakistan; ^4^Instituto de Farmacia, Universidad Austral de Chile, Isla Teja Campus, Valdivia (5090000), Chile; ^5^Instituto de Investigación en Ciencias de la Alimentación-CIAL (CSIC-UAM), C/Nicolás Cabrera, 9, 28049 Madrid, Spain

## Abstract

Antimicrobial resistance is the key threat to global health due to high morbidity and mortality. The alteration of bacterial proteins, enzymatic degradation, and change of membrane permeability towards antimicrobial agents are the key mechanisms of antimicrobial resistance. Based on the current condition, there is an urgent clinical need to develop new drugs to treat these bacterial infections. In the current study, the binding patterns of selected antimicrobial peptides (AMPs) with different multidrug-resistant bacterial strains have been analyzed. Among ten selected AMPs in this study, napin and snakin-1 exhibited the best scores and binding patterns. Napin exhibited strong interactions with penicillin-binding protein 1a of *Acinetobacter baumannii* (with a binding score of -158.7 kcal/mol and ten hydrogen bonds), with glucose-1-phosphate thymidylyltransferase of *Mycobacterium tuberculosis* H37Rv (with a binding score of -107.8 kcal/mol and twelve hydrogen bonds), and with streptomycin 3^″^-adenylyltransferase protein of *Salmonella enterica* (with a binding score of -84.2 kcal/mol and four hydrogen bonds). Similarly, snakin-1 showed strong interactions with oxygen-insensitive NADPH nitroreductase of *Helicobacter pylori* (with a binding score of -105.0 kcal/mol and thirteen hydrogen bonds) and with penicillin-binding protein 2a of methicillin-resistant *Staphylococcus aureus* (with a binding score of -103.8 kcal/mol and twenty-three hydrogen bonds). The docking results were further validated by molecular dynamics simulations. The results of this computational approach support the evidence of efficiency of these AMPs as potent inhibitors of these specific proteins of bacterial strains. However, further validations are required to fully evaluate the potential of selected AMPs as drug candidates against these resistant bacterial strains.

## 1. Introduction

The excessive or inappropriate use of antibiotics has pushed the world towards the postantibiotic era. The bacterial strains mastered their own antidote that has led to severe resistance against many antimicrobial agents [1]. As the multidrug-resistant strains evolve, the invasive infections in hospitals and communities increase day by day in a complex pattern. Many antibiotics are failing now because of the occurrence of bacterial resistance due to mutational changes in the bacterial cellular machinery. Therefore, there is a dire need for potent antimicrobial agents which are less toxic and more effective. From the past few years, antimicrobial peptides (AMPs) have drawn much attention due to their vast therapeutic properties with fewer side effects [2].

AMPs are the first line of defense in plant innate immunity to protect them against microbial infections [3]. In contrast, some bacterial species also produce AMPs to counter other bacterial species in the amensalism relationship to compete and kill other bacterial strains for the same ecological niche [4]. About 17 families of AMPs have been reported with different antibacterial, antifungal, and antiviral activities [5]. These include defensin (PR-12 family), hevein-like peptide, thionin (PR-13 family), knottin, *α*-hairpinin, lipid transfer protein (LTP; PR-14 family), and snakin [6].

AMPs are classified as broad-spectrum antimicrobial agents that can regulate the innate immune system of various organisms such as bacteria, protozoa, fungi, plants, insects, and animals [7]. AMPs exhibit considerable structural and functional diversity that upholds their antimicrobial activity, microbial cell selectivity, and immunomodulatory properties which make them suitable drug candidates for the development of new therapies [8]. After microbial invasion into the host, the AMPs undergo genetic modifications for their expression or rapid transcription [9]. Being conserved in nature, these natural AMPs show specific resistance against certain pathogens including bacteria, pathogenic fungi, viruses, and parasites that invade into the metabolic machinery of the cell [10]. AMPs have been reported from almost all organisms, but more specifically, plant-derived antimicrobial peptides have been reported with structural and functional diversity [11]. Different studies have highlighted the physiological and therapeutic importance of various AMPs against multidrug-resistant pathogens [12]. The antimicrobial activity of AMPs makes them interestingly special as potential drug candidates against various pathogens [2].

Considering the current increase of antibiotic resistance in bacterial species because of their evolution, the discovery of new and effective natural compounds which could be employed in the treatment of various bacterial infections with fewer or no side effects compared to present antibiotics has become important in order to ensure the health of our future generations [13]. The current century witnesses an extraordinary advancement in the field of drug discovery because of the advent of *in silico* approaches of bioinformatics, molecular docking, and molecular dynamics simulation [14]. The advantage of the docking approach over traditional drug discovery is the predictions of protein pairs which enhance our knowledge about biological pathways by examining protein-peptide, protein-protein, or protein-ligand complexes which provide insights into the mechanisms of novel interactions [15].

The bacterial strains *A*. *baumannii*, *M*. *tuberculosis*, *H*. *pylori*, MRSA, and *S*. *enterica* used in this study have been reported by the World Health Organization (WHO) as the most deadly bacteria due to their multidrug resistance [16]. Ten efficient AMPs were selected from the literature and docked against five selected deadly bacterial strains. The purpose of this study was to explore the antibacterial activities of the most reported AMPs against five selected multidrug-resistant bacterial strains. The reported AMPs in this study are assumed to be useful for drug discovery professionals to check their bactericidal and/or bacteriostatic potentials in controlling these bacterial species. To provide a more meaningful *in vivo* prediction of efficacy of these AMPs, it is also necessary to combine the results and information of this study with pharmacokinetic and pharmacodynamic data because the clinical outcome must be the ultimate guide for curing any infection.

## 2. Materials and Methods

### 2.1. Selection and Retrieval of Antimicrobial Peptides

On the basis of reported antimicrobial activities in the literature, ten AMPs were selected for analyses against deadly pathogenic bacterial species. The 3D structures of selected peptides were downloaded from Protein Data Bank (https://www.rcsb.org/) in .pdb format. The selected AMPs and their PDB IDs were as follows: napin (PDB ID: 1PNB), snakin-1 (PDB ID: 5E5Q), knot1 domain-containing protein (PDB ID: 7C31), *Amaranthus caudatus*-AMP2 (PDB ID: 1MMC), EcAMP1 (PDB ID: 2L2R), Nigellin-1.1 (PDB ID: 2NB2), plant defensin NsD7 (PDB ID: 5KK4), flower-specific gamma-thionin (PDB ID: 6DMZ), acyclotide ribe 31 (PDB ID: 7KPD), and antimicrobial peptide 1a (PDB ID: 2LB7).

### 2.2. Retrieval of Receptor Proteins of Pathogenic Bacterial Species

For the current study, the five most virulent bacterial strains were selected. The 3D structures of receptor proteins of selected bacterial strains were downloaded from PDB in .pdb format. The selected proteins with their PDB IDs used in this study as receptor proteins were as follows: penicillin-binding protein 1a of *Acinetobacter baumannii* (PDB ID: 3UDF), glucose-1-phosphate thymidylyltransferase of *Mycobacterium tuberculosis* (PDB ID: 6B5E), oxygen-insensitive NADPH nitroreductase of *Helicobacter pylori* (PDB ID: 3QDL), penicillin-binding protein 2a of methicillin-resistant *Staphylococcus aureus* (PDB ID: 1MWT), and streptomycin 3^″^-adenylyltransferase of *Salmonella enterica* (PDB ID: 6FZB).

### 2.3. Protein-Protein Docking

The hosts produce specific immune responses upon any pathogenic attack. Therefore, to observe the binding patterns and interactions between selected AMPs and specific bacterial receptor proteins, HADDOCK v.2.4 was used to carry out the docking analysis [17]. The educational version of the PyMOL Molecular Graphics System was used to predict the active site of each receptor protein and to visualize and draw interactions between AMPs and receptor proteins [18]. Besides, PDBsum was used to validate the interactions of amino acid residues involved in the docked complex [19]. Chain A of each receptor protein was used in docking studies.

### 2.4. Molecular Dynamics Simulation

Molecular dynamics (MD) simulations were performed for 100 nanoseconds using Desmond, a package of Schrödinger LLC. The initial stages of protein and peptide complexes for molecular dynamics simulation were obtained from docking studies. Molecular docking studies provide a prediction of binding status in static conditions. Simulations were carried out to predict the binding status in the physiological environment. The protein-peptide complexes were preprocessed using Protein Preparation Wizard or Maestro, which also include optimization and minimization of complexes. All systems were prepared by the System Builder tool. A solvent model with an orthorhombic box was selected as TIP3P (Transferable Intermolecular Interaction Potential 3 Points). The OPLS_2005 force field was used in the simulation. The models were made neutral by adding counter ions where needed. To mimic the physiological conditions, 0.15 M salt (NaCl) was added. The NPT ensemble with 300 K temperature and 1 atm pressure was selected for complete simulation. The models were relaxed before the simulation. The trajectories were saved after every 100 ps for analysis, and the stability of simulations was evaluated by calculating the root mean square deviation (RMSD) of the protein and the ligand over time. The Desmond simulation trajectories were analyzed. RMSD, root mean square fluctuation (RMSF), and protein-ligand contacts were calculated from MD trajectory analysis.

## 3. Results and Discussion

Protein-protein interactions play vital roles in cellular activities as these hold the major tasks of the biological machinery. These complex (e.g., protein-peptide complex or protein-ligand complex) interactions help to sort out the mysterious signaling and pathways related to the functioning of living systems. To decode the possible interactions of AMPs against bacterial receptor proteins, computer-mediated molecular docking was performed. HADDOCK server v.2.4 was used to predict the possible protein-protein interactions between AMPs and pathogenic bacterial receptor proteins.

Medicinal plants have a long history in both the traditional and modern medicines in different communities across the world. The crude extract and herbal decoction of many plants have been reported with biologically active compounds that play a significant role in the treatment of many diseases [20]. Recent studies on natural flora have shown the presence of biologically active peptides and phytochemicals with reported therapeutic activities [21]. Proteins are the workhorse of cells with diverse cellular functions as these serve as messengers, modifiers, scaffolds, catalysts, and signal receptors. Protein interactions with other proteins, DNA, RNA, and peptides are responsible for various biological activities [22].

Among ten selected AMPs, only two peptides (i.e., napin and snakin-1) showed the best binding interactions and HADDOCK scores against selected pathogenic bacterial species. The peptide-protein complexes with the lowest binding energy were considered to be the most stable ones (Table 1). Napin showed strong interactions and hydrogen bonding with proteins of three bacterial strains (i.e., *Acinetobacter baumannii*, *Mycobacterium tuberculosis*, and *Salmonella enterica*) while snakin-1 binds with the interacting residues of two bacterial proteins (i.e., *Helicobacter pylori* and methicillin-resistant *Staphylococcus aureus*).

### 3.1. Interactions between AMPs and PBP1a of *Acinetobacter baumannii*

*Acinetobacter baumannii* is a Gram-negative bacterium and has been listed in the group of ESKAPE pathogens which are responsible for a variety of infections, most commonly respiratory and urinary tract infections. The lethality and prevalence of *Acinetobacter* have increased due to resistance against different antibiotics [23]. Over the last three decades, this bacterium has acquired resistance against antibiotics due to adaptational changes in enzymes and cellular proteins [24]. The mortality rate of *Acinetobacter* outbreaks was 50-65% as most of the patients died within 48 hours of hospitalization [25].

**Figure 1:**
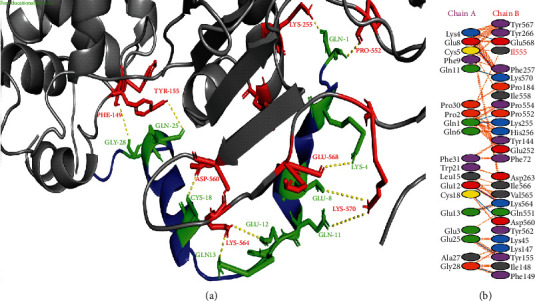
Protein-protein interactions between napin and penicillin-binding protein 1a of *A*. *baumannii*. (a) PBP1a is represented in grey color with red interacting residues, and napin is shown in blue color with green interacting residues. (b) All interacting residues between napin and PBP1a of *A*. *baumannii* complex; hydrogen bonds are shown in blue color, and salt bridges are represented by red-colored lines. Other representing properties of amino acids are represented by different colors (positive: blue, neutral: green, negative: red, aliphatic: grey, Pro&Gly: orange, aromatic: pink, and Cys: yellow).

Penicillin-binding proteins are diverse bifunctional enzymes and classified as PBP1a and PBP1b that perform the assembly of the bacterial cell wall. Different *β*-lactam antibiotics disrupt the bacterial cell wall synthesis by covalently inactivating the penicillin-binding proteins. Access to these periplasmic targets can be helpful to inhibit bacterial activity and appeared as an emerging step in meeting the new challenges represented by multidrug-resistant bacteria [26]. In this study, napin with a HADDOCK score of -158.7 kcal/mol showed strong interactions with active amino acids of penicillin-binding protein 1a of *A*. *baumannii*. The protein is highlighted as grey color with highlighted red sticks as interactive amino acid residues in Figure 1. In a study, thirty-four compounds were tested using a molecular docking approach against PBP1a of *Acinetobacter baumannii*, and neogrifoline and 3,11-dioxolanosta-8,24(Z)-diene-26-oic acid exhibited the best results [13] and could be potential drug candidates against this bacterial species. In another study, Skariyachan et al. [27] used herbal-based ligands to predict receptor-ligand interactions by molecular docking. They revealed that the herbal ligand imipenem exhibited a binding energy of −5.9 kcal/mol when docked with PBP1a and suggested that the lead compound and the target could be used for structure-based drug designing against *A*. *baumannii*.

### 3.2. Interactions between AMPs and RmlA of *Mycobacterium tuberculosis*

*Mycobacterium tuberculosis* is a facultative intracellular pathogen and causative agent of tuberculosis. It has remained one of the main causes of increased mortality and morbidity with approximately two million deaths worldwide [28]. Tuberculosis (TB) is known as a major threat to humanity for the past three decades due to the emergence of multidrug-resistant strains of this bacterium [29]. The cell wall of *M*. *tuberculosis* consists of three interconnected molecules including arabinogalactan, mycolic acids, and peptidoglycan which are responsible for bacterial cell viability [30]. The arabinogalactan is linked to the sixth position of the muramic acid residue of peptidoglycan *via* disaccharide linker *α*-l-rhamnosyl-(1 → 3)-*α*-d-*N*-acetylglucosaminosyl-1-phosphate [31].

**Figure 2:**
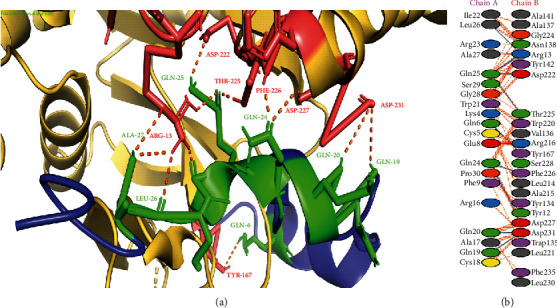
Protein-protein interactions between napin and glucose-1-phosphate thymidylyltransferase of *M*. *tuberculosis* H37Rv. (a) Glucose-1-phosphate thymidylyltransferase is represented in yellow-orange color with deep salmon-colored interacting residues, and napin is shown in blue color with green interacting residues. (b) All interacting residues between napin and glucose-1-phosphate thymidylyltransferase protein of *A*. *baumannii* complex; hydrogen bonds are shown in blue color, and salt bridges are represented by red-colored lines. Other representing properties of amino acids are represented by different colors (positive: blue, neutral: green, negative: red, aliphatic: grey, Pro&Gly: orange, aromatic: pink, and Cys: yellow).

The rhamnose is the precursor in the rhamnosyl biosynthesis pathway not only in *M*. *tuberculosis* but also in a wide range of bacterial species [28]. Therefore, rhamnose plays a crucial role in the attachment of arabinogalactan to peptidoglycan in the bacterial cell wall. Glucose-1-phosphate thymidylyltransferase (RmlA) serves as the leading enzyme of the rhamnose biosynthesis pathway and is therefore essential for the survival of *M*. *tuberculosis* [32]. The glucose-1-phosphate thymidylyltransferase (RmlA) also plays an essential role in bacterial cell wall viability, and therefore, RmlA could serve as a major target in the prevention of this infection. In this study, we focused on protein-protein docking between selected AMPs and glucose-1-phosphate thymidylyltransferase of *M*. *tuberculosis* to explore the potential of AMPs against RmlA. Among ten AMPs, napin with a score of -107.8 kcal/mol showed twelve hydrogen bonds with glucose-1-phosphate thymidylyltransferase of *M*. *tuberculosis*. The interactions have been shown in Figure 2. Mansuri et al. [32] docked two compounds (i.e., 6-[(2R,3S,5R)-5-[5-(2-aminoethyl)-2,4-dioxo-1,2,3,4-tetrahydropyrimidin-1-yl]-3-hydroxyoxolan-2-yl] hexanoic acid and 4-(2-{1-[(1S,3S,4S)-3-(5-carboxypentyl)-4-hydroxy-2-methylidenecyclopentyl]-2,4-dioxo-1,2,3,4-tetrahydropyrimidin-5-yl}ethyl)morpholin-4-ium) against glucose-1-phosphate thymidylyltransferase of *M*. *tuberculosis* and reported that these compounds could be used as competitive inhibitors.

### 3.3. Interactions between AMPs and Streptomycin 3^″^-Adenylyltransferase of *Salmonella enterica*

*Salmonella enterica* is a facultative Gram-negative intracellular bacterium that infects both animals and humans. Growing pieces of evidence have pointed towards more severity of infections due to the resistant strains of *S*. *enterica* [33]. The streptomycin and spectinomycin belong to aminocyclitol and aminoglycoside families of antibiotics, respectively, and bind to the bacterial ribosome and interfere with the protein biosynthesis.

**Figure 3:**
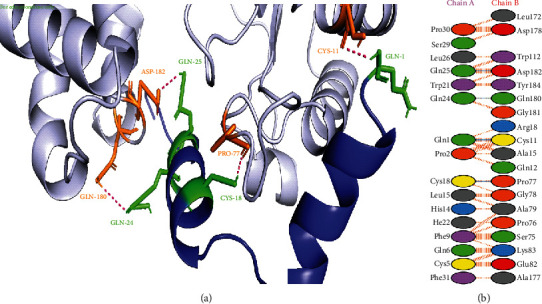
Protein-protein interactions between napin and streptomycin 3^″^-adenylyltransferase protein of *S*. *enterica*. (a) Streptomycin 3^″^-adenylyltransferase is represented in light blue color with orange-colored interacting residues, and napin is shown in blue color with green interacting residues. (b) All interacting residues between napin and streptomycin 3^″^-adenylyltransferase protein of *A*. *baumannii* complex; hydrogen bonds are shown in blue color, and salt bridges are represented by red-colored lines. Other representing properties of amino acids are represented by different colors (positive: blue, neutral: green, negative: red, aliphatic: grey, Pro&Gly: orange, aromatic: pink, and Cys: yellow).

Currently, the most reported resistance in *S*. *enterica* is due to the inactivation of these drugs by aminoglycoside-modifying enzymes such as aminoglycoside nucleotidyltransferases (ANTs). Aminoglycoside (3^″^) (9) adenylyltransferase AadA from *S*. *enterica* is the member of the ANT(3^″^)-Ia family that *O*-adenylates the streptomycin and spectinomycin at specific positions. Thus, AadA is the promising target to hinder the protein machinery of this bacterium [34]. In this study, napin showed the best binding patterns with streptomycin 3^″^-adenylyltransferase protein of *S*. *enterica* with a score of -84.2 kcal/mol and only four hydrogen bonds (Figure 3). The HADDOCK server predicts and gives different numbers of hydrogen bonds for different protein-peptide complexes as it depends on how strongly the peptide is interacting with the receptor protein. In a study, Prabhu et al. [35] used molecular docking and molecular dynamics simulation approaches to predict structural, binding, and pharmacokinetic properties of different selected compounds. They docked these compounds against streptomycin 3^″^-adenylyltransferase of *Serratia marcescens* and reported that the best five identified compounds could be used as potential drug entities to develop antipathogenic agents.

### 3.4. Interactions between AMPs and Oxygen-Insensitive NADPH Nitroreductase of *Helicobacter pylori*

**Figure 4:**
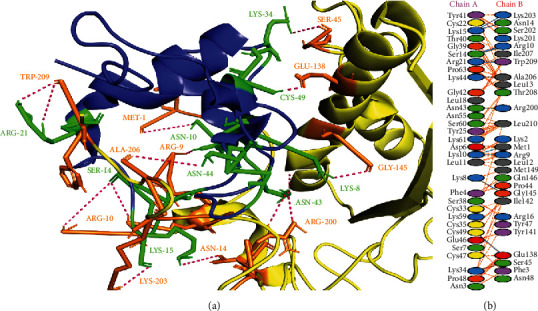
Protein-protein interactions between snakin-1 and oxygen-insensitive NADPH nitroreductase of *H*. *pylori*. (a) NADPH nitroreductase is represented in yellow color with orange-colored interacting residues, and snakin-1 is shown in blue color with green interacting residues. (b) All interacting residues between snakin-1 and NADPH nitroreductase protein of *A*. *baumannii* complex; hydrogen bonds are shown in blue color, and salt bridges are represented by red-colored lines. Other representing properties of amino acids are represented by different colors (positive: blue, neutral: green, negative: red, aliphatic: grey, Pro&Gly: orange, aromatic: pink, and Cys: yellow).

*Helicobacter pylori* is a microaerophilic Gram-negative bacterium that colonizes the gastric mucosa. *H*. *pylori* is one of the world's most common pathogens affecting about 50% of the world's population. This bacterium affects the human gastrointestinal tract and causes upper gastrointestinal tract infections such as chronic gastritis, ulcerative colitis, gastrointestinal/mucosa-associated lymphoid tissue (MALT) lymphoma, duodenal ulcer, and gastric carcinoma [36]. The frequent consumption and high dosage of the metronidazole (MTZ) antibiotic are responsible for resistance in the bacterium with a negative impact on the treatment efficacy. The mutations in the *rdxA* gene (oxygen-insensitive) and *frxA* gene (flavin reductase) that encode NADPH nitroreductases have been associated with the onset of metronidazole resistance by *H*. *pylori* [37]. Snakin-1 with a score of -105.0 kcal/mol showed 13 hydrogen bonds with oxygen-insensitive NADPH nitroreductase of *H*. *pylori*. The interactions have been displayed in Figure 4. In a study, Mulimani et al. [38] used 100 compounds in a molecular docking study against oxygen-insensitive NADPH nitroreductase of *H*. *pylori* and reported that benzimidazoles and oxacillin exhibited the best results and therefore could be used as potential inhibitors.

### 3.5. Interactions between AMPs and PBP2a of MRSA

*Staphylococcus aureus* is a Gram-positive pathogen that is capable of spreading a wide spectrum of infections. Different strains of *S*. *aureus* have been isolated in the past which are resistant to multiple drugs and responsible for the severe outbreaks of infections worldwide. Among all the isolated strains, MRSA is notably known as a resistant strain due to the unique genetic element staphylococcal chromosomal cassette mec (SCCmec) that carries the mecA gene which encodes penicillin-binding protein 2a (PBP2a) [39]. PBP2a is the product of the mutant gene which plays a role as surrogate transpeptidase in the absence of other PBPs [40].

**Figure 5:**
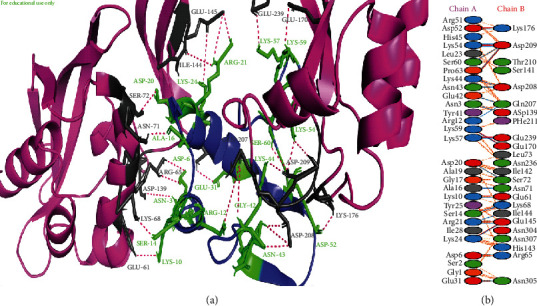
Protein-protein interactions between snakin-1 and penicillin-binding protein 2a from methicillin-resistant *Staphylococcus aureus*. (a) PBP2a is represented in pink color with grey-colored interacting residues, and snakin-1 is shown in blue color with green interacting residues. (b) All interacting residues between snakin-1 and PBP2a protein of *A*. *baumannii* complex; hydrogen bonds are shown in blue color, and salt bridges are represented by red-colored lines. Other representing properties of amino acids are represented by different colors (positive: blue, neutral: green, negative: red, aliphatic: grey, Pro&Gly: orange, aromatic: pink, and Cys: yellow).

*S*. *aureus* (MRSA) manifests the resistance to methicillin and other *β*-lactams due to the production of PBP2a that exhibits transpeptidase activity for cell wall biosynthesis [39]. Hence, there is a dire need for new drugs to inhibit the biosynthesis of the cell wall of *S*. *aureus*. In this study, snakin-1 showed the best binding pattern with a score of -103.8 kcal/mol and exhibited twenty-three hydrogen bonds with PBP2a of *S*. *aureus* (MRSA). The detail has been discussed in cartoon representation with highlighted portions in Figure 5. In a study, Murugavel et al. [41] revealed the inhibitory activity of methyl (2E)-2-{[N-(2-formylphenyl) (4-methylbenzene)sulfonamido]methyl}-3-(4-chlorophenyl) prop-2-enoate (MFMSC) when docked against PBP-2X. Similarly, Levy et al. [42] docked the compound trans 2-(aminomethyl)-4-oxazol-5-yl-7-oxo-1,6-diazabicyclo[3.2.1]oct-3-en-6-yl] hydrogen sulfate (CPD4) against PBP2 of *Escherichia coli* and reported that CPD4 could be a potential scaffold for the development of active molecules which would be effective against a broad range of bacterial species.

### 3.6. Molecular Dynamics Simulation

**Figure 6:**
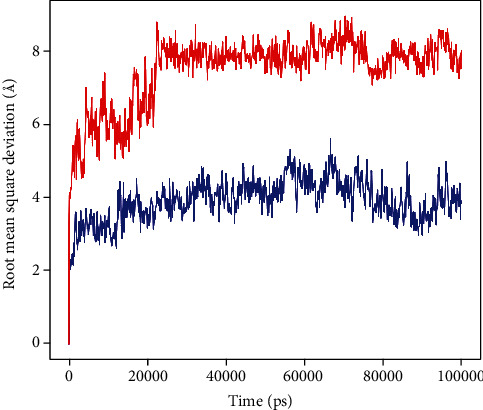
Root mean square deviation (RMSD) of penicillin-binding protein 1a of *A*. *baumannii* (protein) and napin (peptide) with time. The left *Y*-axis shows the variation of protein RMSD through time. Peptide RMSD is shown in red, and protein RMSD is shown in blue.

**Figure 7:**
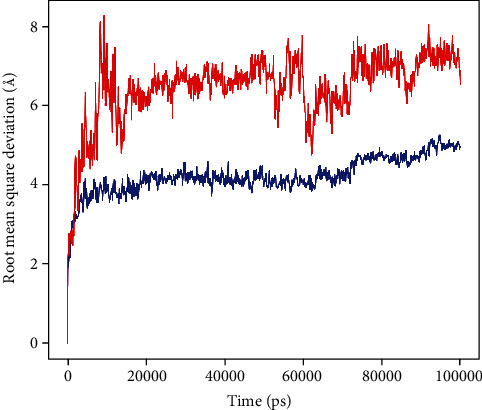
Root mean square deviation (RMSD) of oxygen-insensitive NADPH nitroreductase (protein) of *H*. *pylori* and snakin-1 (peptide) with time. The left *Y*-axis shows the variation of protein RMSD through time. Peptide RMSD is shown in red, and protein RMSD is shown in blue.

Based on the best HADDOCK scores, two complexes (i.e., napin with PBP1a of *A*. *baumannii* and snakin-1 with oxygen-insensitive NADPH nitroreductase of *H*. *pylori*) were selected for MD simulation studies. The evolution of RMSD values in the course of time for the C-alpha atoms of the peptide-bound proteins is shown in Figures 6 and 7. The RMSD plot of the complex Napin-PBP indicated that the complex reached stability at 20 ns, and the RMSD plot of the complex Snakin-NADPH indicated that the complex reached stability at 5 ns. From then, an average RMSD value of 1.5 Å for Napin-PBP and 1.0 Å for Snakin-NADPH persists up to 100 ns during the simulation period, which is quite acceptable. Peptides fit to proteins, and RMSD values fluctuated within 2.5 Å after being stable. These indicate that the peptides remained stably bound to the binding sites of their respective receptors during the simulation period. However, there is more deviation in Napin-PBP compared to Snakin-NADPH.

**Figure 8:**
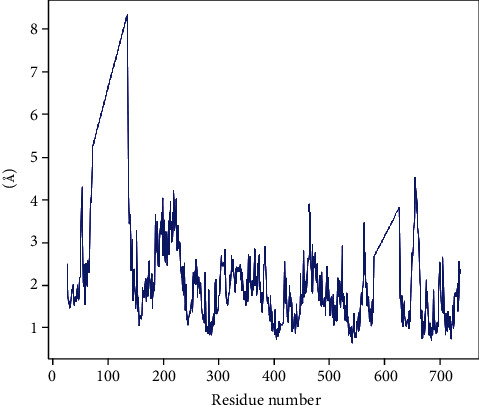
Residue-wise root mean square fluctuation (RMSF) of protein (penicillin-binding protein 1a of *A*. *baumannii*) and peptide (napin) (PBP1a-napin complex).

**Figure 9:**
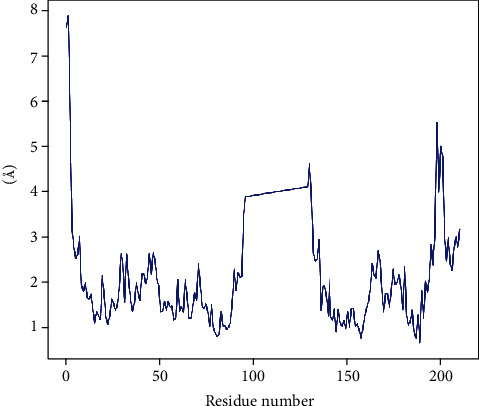
Residue-wise root mean square fluctuation (RMSF) of protein (oxygen-insensitive NADPH nitroreductase of *H*. *pylori*) and peptide (snakin-1) (NADPH-snakin-1 complex).

The residue-wise RMSF values of proteins bound to their respective peptides are shown in Figures 8 and 9. The residues are showing higher peaks corresponding to loop regions, as identified from MD trajectories (Figure S1 and S2), or N- and C-terminal zones. Low RMSF values of binding site residues indicate the stability of the peptide binding with the protein.

**Figure 10:**
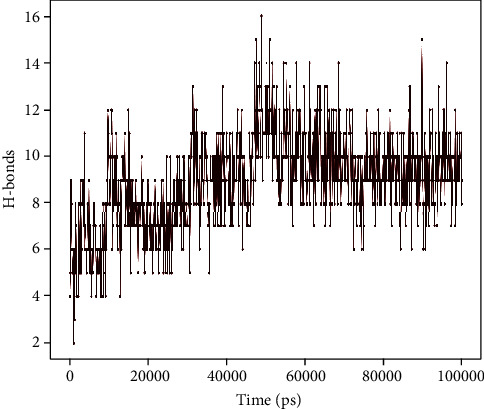
Protein-peptide contact histogram of penicillin-binding protein 1a of *A*. *baumannii* and napin complex.

**Figure 11:**
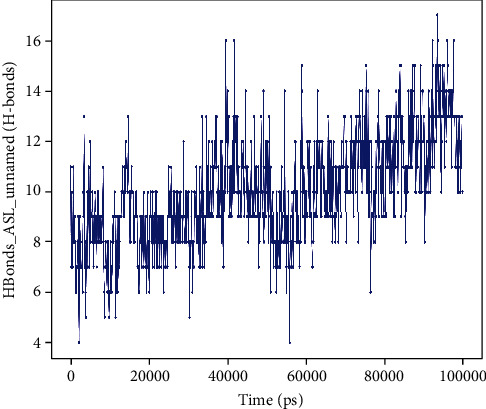
Protein-peptide contact histogram of oxygen-insensitive NADPH nitroreductase of *H*. *pylori* and snakin-1 complex.

Most of the important interactions of protein-peptide determined with MD are hydrogen bonds, as depicted in Figures 10 and 11.

*In silico* studies on pharmacological and nutraceutical applications of plant-derived compounds are more efficient and less laborious [43]. Molecular docking is an elaborative method which helps scientists to forecast the best binding patterns and interactions between desired compounds before the experimental and laboratory approaches [44]. Computational biology applies different coherent and integrated approaches to analyze and explore new features from large collections of biological data such as whole genomes and proteomes of eukaryotes and prokaryotes [45]. Different docking techniques such as ligand-based molecular docking, protein-protein docking, protein-peptide docking, and induced fit docking have greatly influenced the field of drug discovery. Computer-aided drug designing using molecular docking and molecular dynamics simulation approaches provides rapid screening of novel and potential drug candidates to predict drug-receptor interactions [46]. In recent years, plant-derived natural compounds have been proved as potent drug candidates and inhibitors of many pathogenic proteins that play crucial roles in the pathogenesis of various diseases [47]. Recently, using computational modeling, the molecular dynamics aspects of moxifloxacin-induced resistance in *M*. *tuberculosis* DNA gyrase A and C have been studied by Pandey et al. [48]. Similarly, Bera et al. [49] also used molecular docking and simulation approaches to study interactions of Echinocandin B with the multidrug resistance-associated protein family of ATP-binding transporter protein.

The alteration of bacterial proteins and membrane permeability are the major reasons for the rapid emergence of bacterial resistance towards antibiotics [50]. The level of antibiotic resistance in bacteria is rising very rapidly due to the emergence of new bacterial strains. Along with modifications in bacterial proteins and enzymes, the second leading cause of the emergence of antibiotic resistance is the behavioral changes and prescriptions of antibiotics [51]. With the continuous use of these drugs against bacterial infections without any valid prescription, the emergence and spread of bacterial resistance would worsen in the coming time [52]. Bacterial resistance towards antibiotics is the main cause of outbreaks of specific infections. The mutations and advanced modifications in the bacterial machinery are the leading causes of antimicrobial resistance. The modified enzymes and mutant genes make bacteria no longer responsive to specific antibiotics. According to WHO, these multidrug-resistant bacteria are known as superbugs because current antibiotics are inefficient against them [53]. On the basis of the current situation, there is an urgent need for new and novel antimicrobial drugs that could inhibit the bacterial machinery directly.

The antibiotics which are used to treat *A*. *baumannii*, *M*. *tuberculosis*, *H*. *pylori*, *S*. *aureus*, and *S*. *enterica* are becoming more limited. Little is known about the contributions of penicillin-binding protein 1a, glucose-1-phosphate thymidylyltransferase, oxygen-insensitive NADPH nitroreductase, penicillin-binding protein 2a, and streptomycin 3^″^-adenylyltransferase which could serve as excellent targets for the development of new antibiotics. In the literature, using molecular docking and simulation studies, the compounds from plant sources have shown better binding with strong interactions, hydrogen bonding, binding energy, and other weak interactions with their normal targets compared to different antibacterial agents which have been used conventionally (e.g., imipenem, polymyxin E, and clinafloxacin) [27]. The aim of this study was therefore to explore the binding interactions between active amino acid residues of AMPs and five different bacterial receptor proteins. This study will help for further validation and exploration of new drugs to cure these infections. The results of this study have proved that these infections are no longer incurable but the treatments might be hidden in some other aspects.

## 4. Conclusion

The current study focuses on the computational prediction of antimicrobial peptides of plant sources against prioritized targets of different bacterial species. Molecular docking and molecular dynamics simulations suggested that among ten peptides, only napin and snakin-1 showed strong binding interactions and the best scores with the selected receptor proteins of bacterial strains. Napin showed noteworthy interactions with three bacterial strains including *A*. *baumannii*, *M*. *tuberculosis*, and *S*. *enterica* while snakin-1 revealed binding interactions with interacting residues of two bacterial proteins such as *H*. *pylori* and methicillin-resistant *Staphylococcus aureus*. Further, the molecular dynamics simulation studies also confirmed that the peptides napin and snakin-1 remained firmly bound to the binding sites of proteins PBP1a of *A*. *baumannii* and oxygen-insensitive NADPH nitroreductase of *H*. *pylori*, respectively, during the simulation period. The findings of this study will help for further advancement and development of new drugs from the natural flora. The aim of this study was to explore the interactive sites and interactions between AMPs and active amino acids of selected bacterial receptor proteins in order to inhibit and target them directly.

## Figures and Tables

**Table 1:** Sources and binding scores (in kcal/mol) of selected AMPs docked against selected receptor proteins of deadly pathogenic bacterial species.

Sr. no.	AMP	Source	*A*. *baumannii*	*M*. *tuberculosis*	*H*. *pylori*	MRSA	*S*. *enterica*
1	Napin	*Brassica napus*	−158.7 ± 12.2	−107.8 ± 14.3	−86.2 ± 4.9	−99.1 ± 9.1	−84.2 ± 9.1
2	Snakin-1	*Solanum tuberosum*	−101.3 ± 8.6	−54.6 ± 6.1	−105.0 ± 9.2	−103.8 ± 6.2	−75.2 ± 4.3
3	Knot1 domain-containing protein	*Vitis vinifera*	−72.7 ± 8.8	−52.0 ± 18.1	−83.1 ± 18.4	−89.8 ± 5.8	−83.6 ± 12.3
4	*Amaranthus caudatus*-AMP2	*Amaranthus caudatus*	−56.6 ± 10.2	−87.0 ± 7.5	−55.2 ± 12.2	−74.0 ± 18.4	−79.0 ± 8.9
5	Antimicrobial peptide EcAMP1	*Echinochloa crus*-*galli*	−124.9 ± 14.0	−44.0 ± 8.5	−98.2 ± 9.6	−98.5 ± 7.0	−79.1 ± 6.8
6	Nigellin-1.1	*Nigella sativa*	−68.8 ± 12.9	−72.2 ± 11.0	−92.8 ± 4.1	−65.9 ± 9.0	−79.0 ± 3.4
7	Plant defensin NsD7	*Nicotiana suaveolens* x *Nicotiana tabacum*	−96.8 ± 9.3	−75.5 ± 10.5	−80.2 ± 15.0	−102.8 ± 19.2	−63.5 ± 12.5
8	Flower-specific gamma-thionin	*Zea mays*	−53.4 ± 10.0	−39.8 ± 21.8	−93.5 ± 14.3	−97.3 ± 24.0	−81.9 ± 10.1
9	Acyclotide ribe 31	*Rinorea bengalensis*	−52.9 ± 10.6	−75.3 ± 4.7	−50.4 ± 1.5	−78.2 ± 2.0	−71.5 ± 18.1
10	Antimicrobial peptide 1a	*Triticum kiharae*	−58.7 ± 1.6	−70.2 ± 16.9	−92.8 ± 30.8	−75.7 ± 7.9	−64.4 ± 4.3

## Data Availability

The data used to support the findings of this study are available from the corresponding author upon request.

## Supplementary Materials

Supplementary Materials Figure S1: protein secondary structure element distribution by the residue index throughout the protein structure (Napin-PBP1a). Red columns indicate *α*-helices, and blue columns indicate *β*-strands. Figure S2: protein secondary structure element distribution by the residue index throughout the protein structure (Snakin-NADPH). Red columns indicate *α*-helices, and blue columns indicate *β*-strands. *(Supplementary Materials)*.
