# Supplementary material for: Investigation of Plant Antimicrobial Peptides against Selected Pathogenic Bacterial Species Using a Peptide-Protein Docking Approach
Source: Biomed Res Int. 2022 Mar 21;2022:1077814. doi: 10.1155/2022/1077814 (PMC8960006; doi:10.1155/2022/1077814)
Supplement: Supplementary Materials — Figure S1: protein secondary structure element distribution by the residue index throughout the protein structure (Napin-PBP1a). Red columns indicate α-helices, and blue columns indicate β-strands. Figure S2: protein secondary structure element distribution by the residue index throughout the protein structure (Snakin-NADPH). Red columns indicate α-helices, and blue columns indicate β-strands. (Supplementary Materials). [file 1077814.f1.docx]

**Investigation of plant antimicrobial peptides against selected pathogenic bacterial species using peptide-protein docking approach**

**Supplementary file**


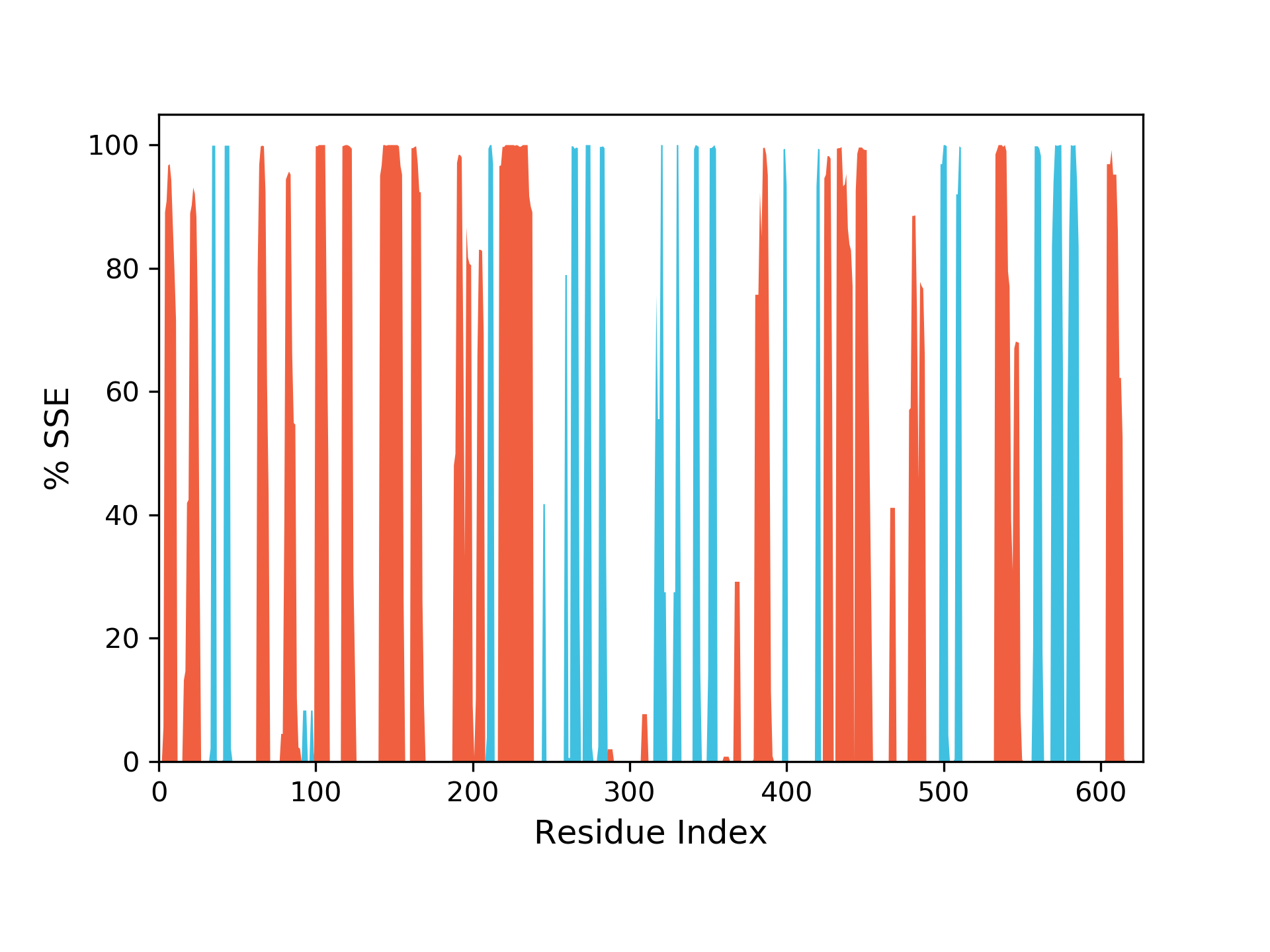


Figure S1: Protein Secondary Structure element distribution by residue index throughout the protein structure (Napin-PBP1a). Red columns indicate α-helices, and blue columns indicate β-strands.


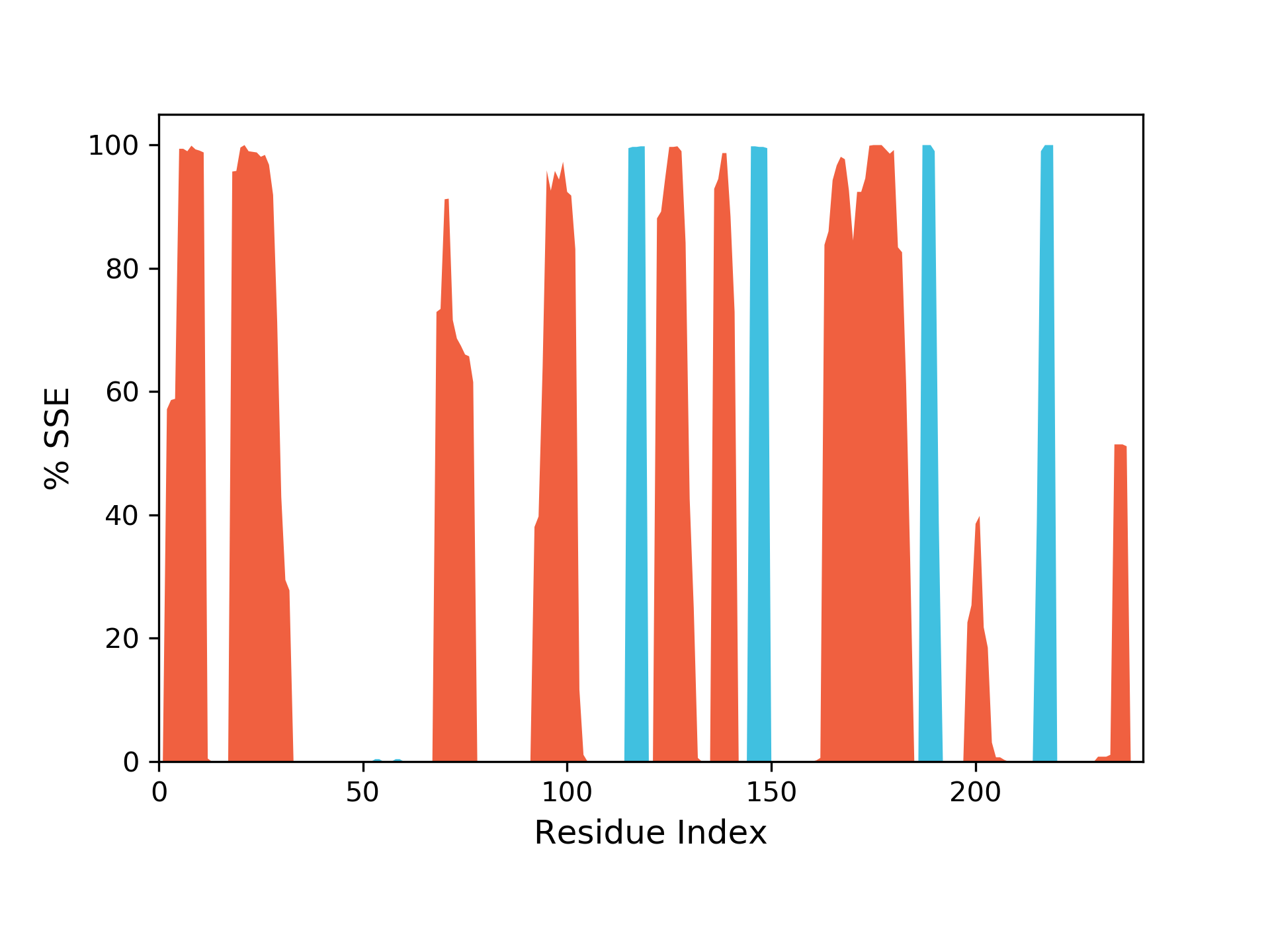


Figure S2: Protein Secondary Structure element distribution by residue index throughout the protein structure (Snakin-NADPH). Red columns indicate α-helices, and blue columns indicate β-strands.
